# Supplementary material for: PilY1 and minor pilins form a complex priming the type IVa pilus in Myxococcus xanthus
Source: Nat Commun. 2020 Oct 7;11:5054. doi: 10.1038/s41467-020-18803-z (PMC7541494; doi:10.1038/s41467-020-18803-z)
Supplement: Supplementary file 1 — Supplementary Information [file 41467_2020_18803_MOESM1_ESM.pdf]

Supplementary Materials for

PilY1 and minor pilins form a complex priming the type IVa pilus in *Myxococcus xanthus*

Anke Treuner-Lange, Yi-Wei Chang, Timo Glatter, Marco Herfurth, Steffi Lindow, Georges Chreifi, Grant J. Jensen & Lotte Søgaard-Andersen*

*Correspondence to: sogaard@mpi-marburg.mpg.de

This file in includes:

1. Supplementary Figures 1-13
2. Supplementary Tables 1-2
3. Supplementary References

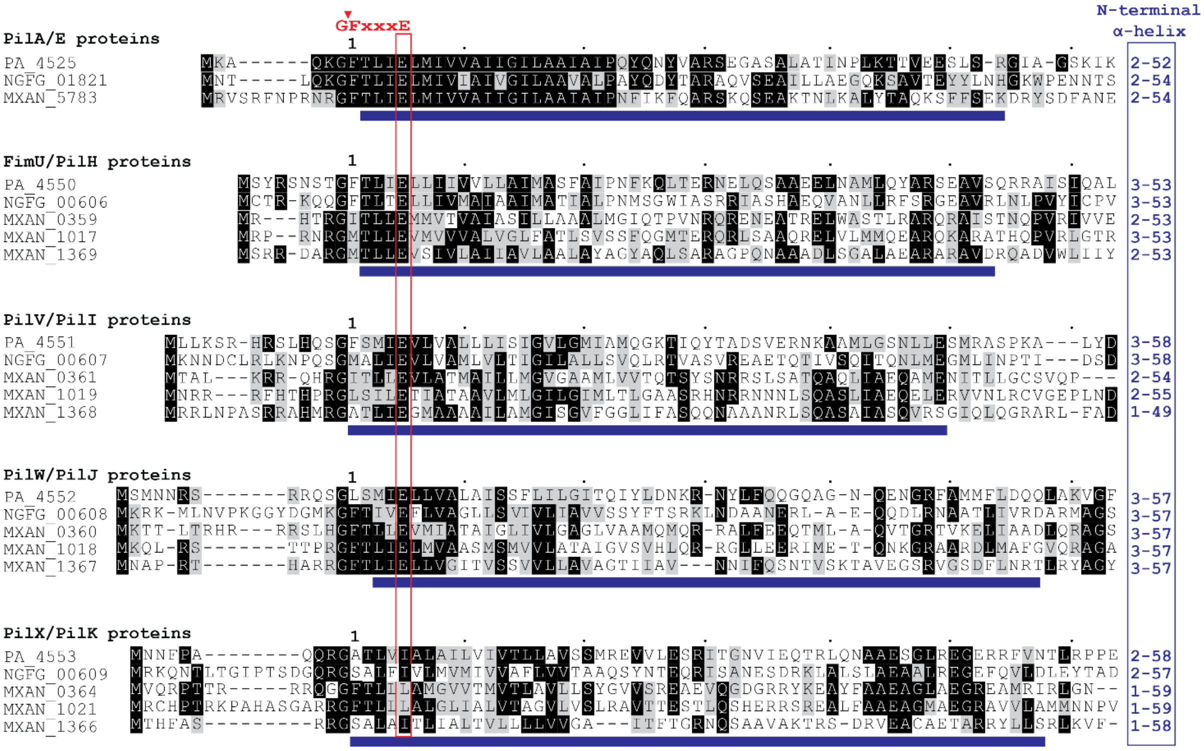

Supplementary Figure 1. Major and minor pilins of *M. xanthus* have a the type III signal peptide and an N-terminal α-helix

N-terminus of major and minor pilins of *M. xanthus*, *P. aeruginosa* and *N. gonorrhoeae* were aligned with T-Coffee using ClustalW output format. The type III signal peptide cleavage site (G↓FxxxE) is shown above in red and the Glu5 residue in the mature proteins is boxed. Note that PilX does not contain the Glu5 residue. The bars below the alignments indicate the predicted N-terminal α-helix for the *M. xanthus* proteins and numbers to the right of the alignment indicate the coordinates of the predicted N-terminal α-helix in the mature proteins. Note that the major pilin in *N. gonorrhoeae* is termed PilE, while minor pilins FimU3, PilV3, PilW3 and PilX3 are named PilH, PilI, PilJ and PilK respectively in *N. gonorrhoeae*.

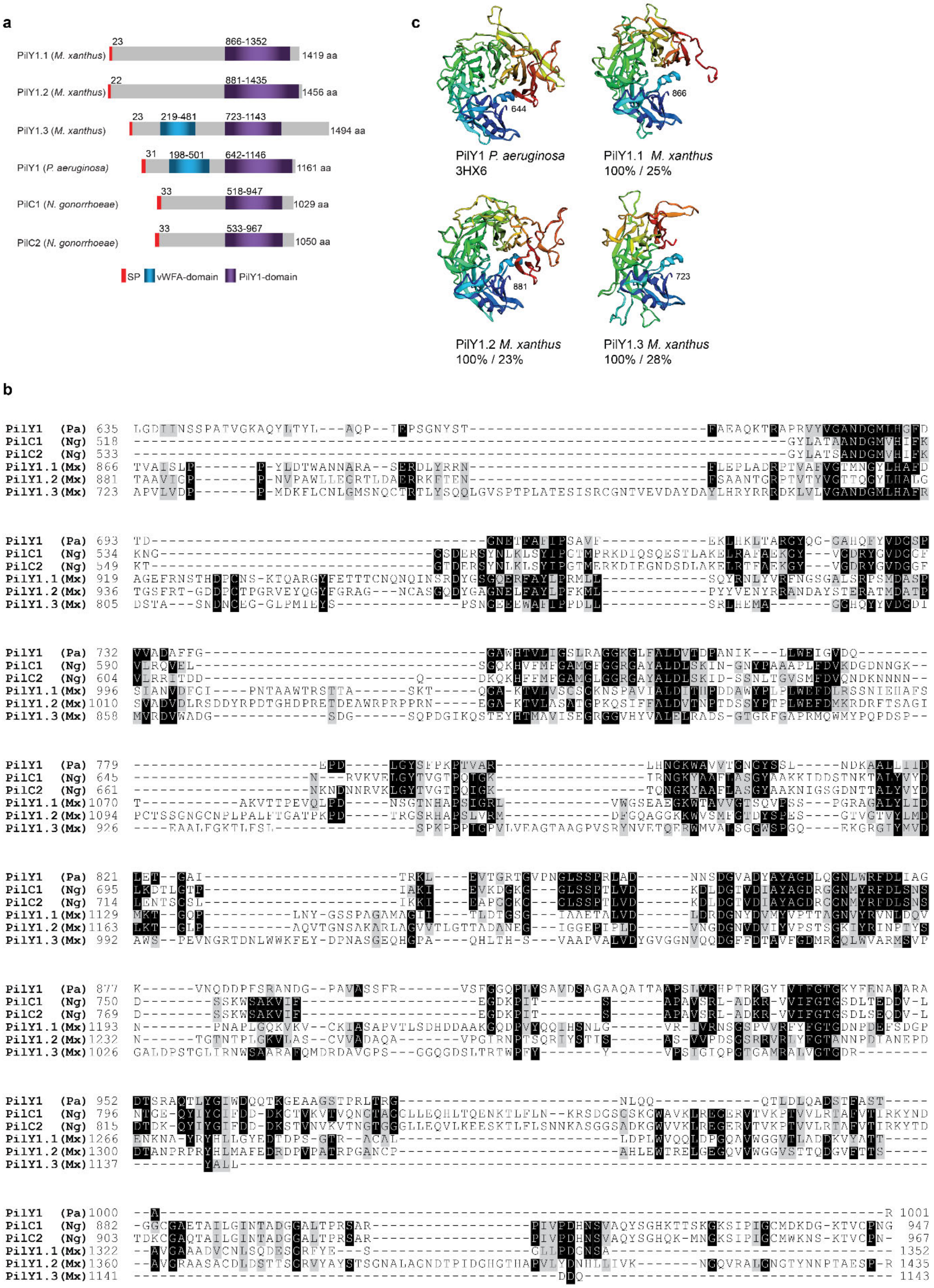

Supplementary Figure 2. *M. xanthus* contains three PilY1 proteins

a Domain architecture of PilY1 proteins of *M. xanthus*, *P. aeruginosa* (PA_4554) and *N. gonorrhoeae* (Z50180, Z49120). The type I signal peptide (SP, red) was predicted using SignalP 4.1 and the conserved PilY1-domain (violet) and von Willebrand Factor A domain (vWFA, blue) were predicted using the PHYRE2 protein fold recognition tool. Numbers at the top left indicate the first residue after the predicted signal peptide cleavage site and numbers above domains represent domain coordinates. **b** Alignment of PilY1 domains of the indicated PilY1 proteins of *M. xanthus*, *P. aeruginosa* and *N. gonorrhoeae* generated with T-Coffee using ClustalW output format. **c** Homology models of conserved PilY1 domain of PilY1 proteins of *M. xanthus.* Rainbow colored structure of the PilY1-domain of PilY1 from *P. aeruginosa* (3HX6) [https://www.wwpdb.org/pdb?id=pdb_00003hx6][1] (N- to C-terminus, blue to red) together with the PHYRE2 homology models based on 3HX6 of the PilY1-domains of the three PilY1 proteins from *M. xanthus.* For each homology model, the confidence/identity level is given in %.

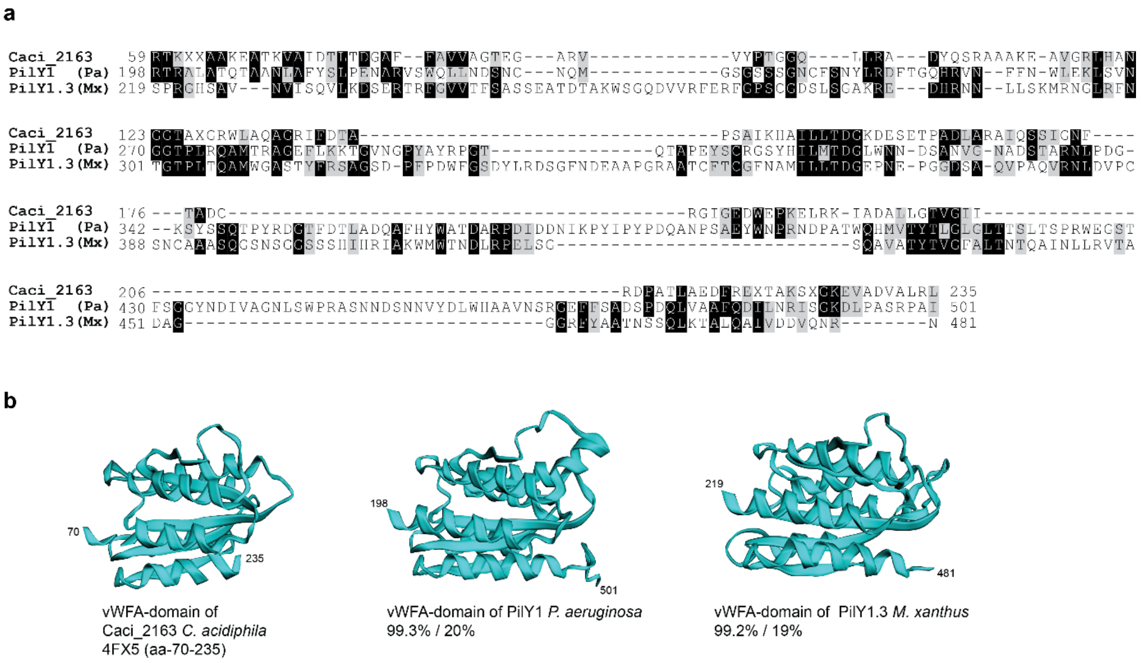

Supplementary Figure 3. Analysis of von Willebrand Factor A (vWFA) domain in PilY1.3

a Alignment of vWFA domains from the indicated proteins. The vWFA domains from the indicated proteins of *Catenulispora acidiphila*, *M. xanthus*, and *P. aeruginosa* were aligned with T-Coffee using ClustalW output format. **b** Homology model of vWFA domains of PilY1.3 of *M. xanthus* and PilY1 from *P. aeruginosa*. PHYRE2 homology models of vWFA domains of PilY1.3 of *M. xanthus* and PilY1 from *P. aeruginosa* were generated based on the vWFA domains of the *C. acidiphila* protein Caci_2163 (4FX5) [https://www.wwpdb.org/pdb?id=pdb_00004fx5]. For each homology model, the confidence/identity level is given in %.

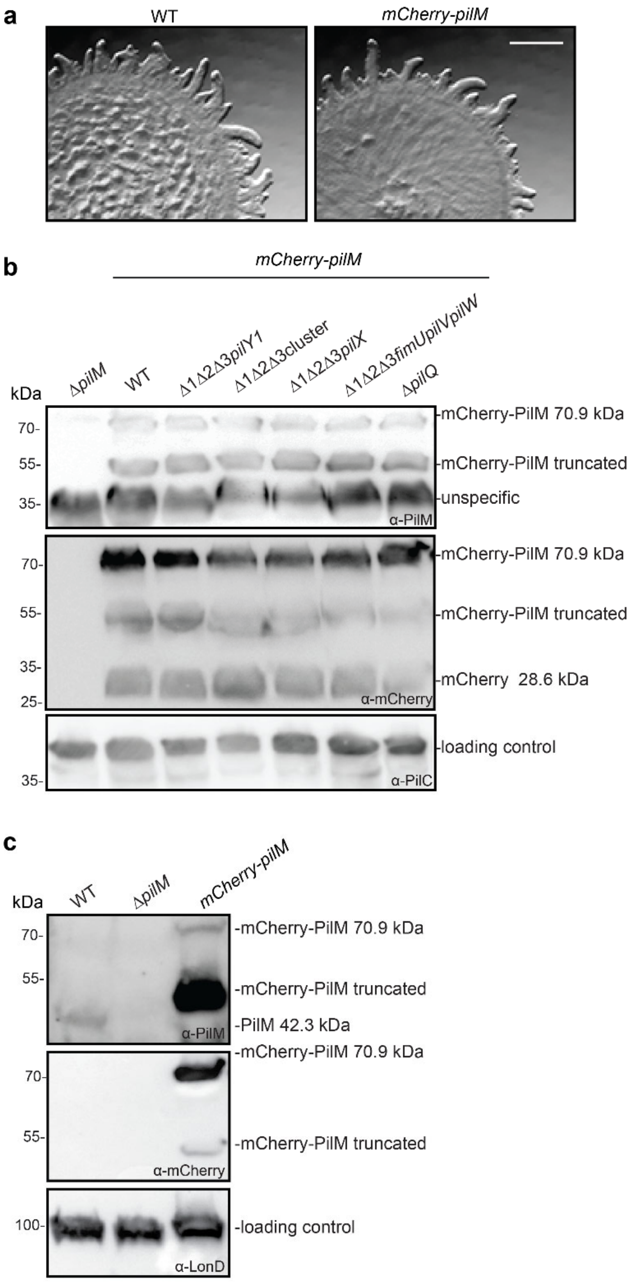

Supplementary Figure 4. The mCherry-PilM fusion is functional
a The mCherry-PilM fusion is functional. The indicated *M. xanthus* strains were incubated at for 24 h. Scale bar, 1 mm. In the *mCherry-pilM* strain, the native *pilM* gene was replaced by *mCherry-pilM*. **b** Accumulation levels of mCherry-PilM. Protein from 3×10^8 cells of the indicated genotypes were loaded per lane and blots probed with α-PilM, α-mCherry and α-PilC antibodies (loading control). Note that the α-PilM antibodies detect an additional band at 35 kDa, which is non-specific as it is also observed in cell extracts of the Δ*pilM* mutant. Also, α-PilM as well as α-mCherry antibodies detect a protein, which is likely a truncated variant of mCherry-PilM. **c** Accumulation level of mCherry-PilM in comparison to native PilM. Protein from 3×10^8 cells were loaded per lane and the blots probed with α-PilM, α-mCherry and α-LonD antibodies (loading control). In order to detect native PilM with the α-PilM antibodies,

SDS-PAGE was done to better separate proteins with a MW between 35 and 70 kDa. Proteins with their calculated molecular masses and positions of molecular markers are indicated. Source data are provided as a Source Data file.

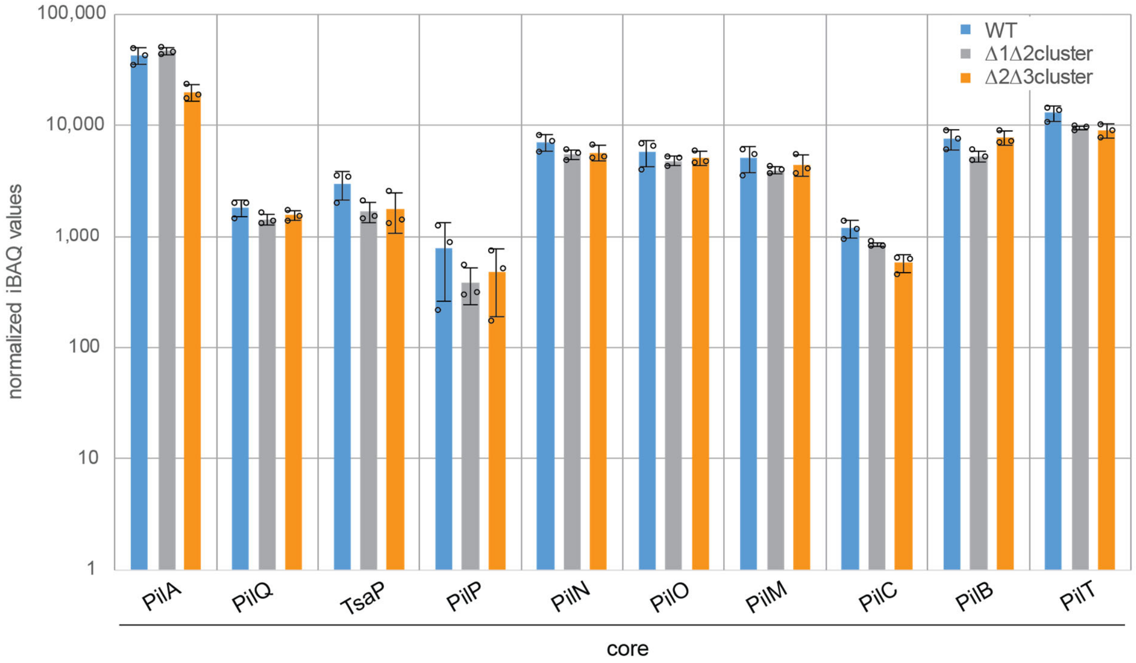

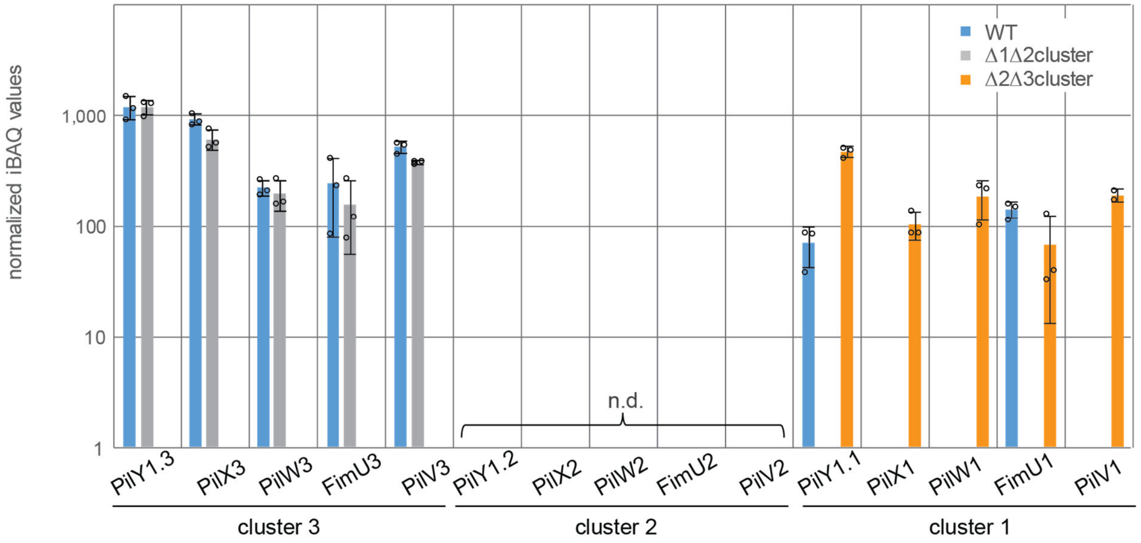

Supplementary Figure 5. Accumulation levels of T4aPM proteins, minor pilins and PilY1 proteins

Strains of the indicated genotypes were grown on 1% CTT 1.5% agar, total cell extract isolated, samples analyzed by LFQ-MS, and mean normalized iBAQ values (absolute values/10,000) calculated. The upper diagram depicts the accumulation levels of the core proteins, while the bottom diagram depicts the accumulation levels of minor pilins and PilY1 proteins of the three clusters. Columns and error bars, mean ± standard deviations from three biological replicates, dots represent the corresponding data points;n.d., not detected. Source data are provided as a Source Data file.

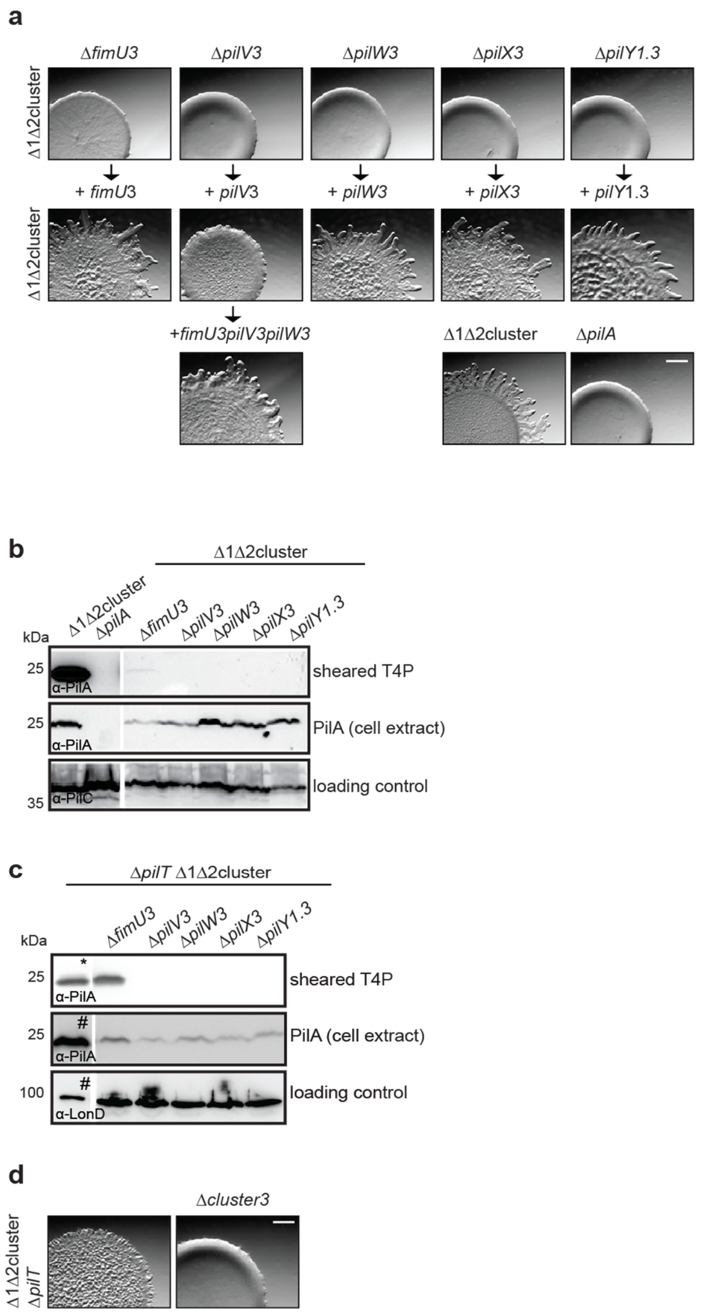

Supplementary Figure 6. Cluster_3 proteins are important for T4aP-dependent motility and T4aP extension

a Effect of single gene deletions in cluster_3 in a Δ1Δ2cluster mutant on T4aP-dependent motility (top row). The indicated *M. xanthus* strains were incubated at for 24 h. Middle row, strains with ectopic expression of the deleted gene from the *pilA* promoter on plasmids integrated in a single copy at the Mx8 *attB* site in the corresponding strain. Bottom row, in the case of the Δ*pilV3* mutant, efficient complementation was only observed upon co-expression with *fimU3* and *pilW3*, the genes *pilV3* is translationally coupled to (Fig. 1b). Control strains are shown to the right in the bottom row. Scale bar, 1 mm. **b** Effect of single gene deletions in cluster_3 in a Δ1Δ2cluster mutant on T4aP formation. Samples were

prepared as in Fig. 1d. α-PilC antibodies were used as a loading control. **c** Effect of additional deletion of *pilT* on T4aP formation in strains with single gene deletions in cluster_3 in a Δ1Δ2cluster mutant. Samples were prepared as in (**b**). Pili as well as total cell extract from a Δ1Δ2 cluster Δ*pilT* mutant have increased levels of PilA. For better comparison, 5% of T4aP sheared from the Δ1Δ2clusterΔ*pilT* strain (*) and 25% of cell extract of the Δ1Δ2clusterΔ*pilT* strain (#) were applied. α-LonD antibodies were used as a loading control. **d** Effect of additional deletion of *pilT* on T4aP-dependent motility in a strain with a deletion of cluster_3 in a Δ1Δ2cluster mutant background. Cells were treated as in (**a**). Gaps between lanes in immunoblots indicate lanes that were deleted for presentation purposes. Source data are provided as a Source Data file.

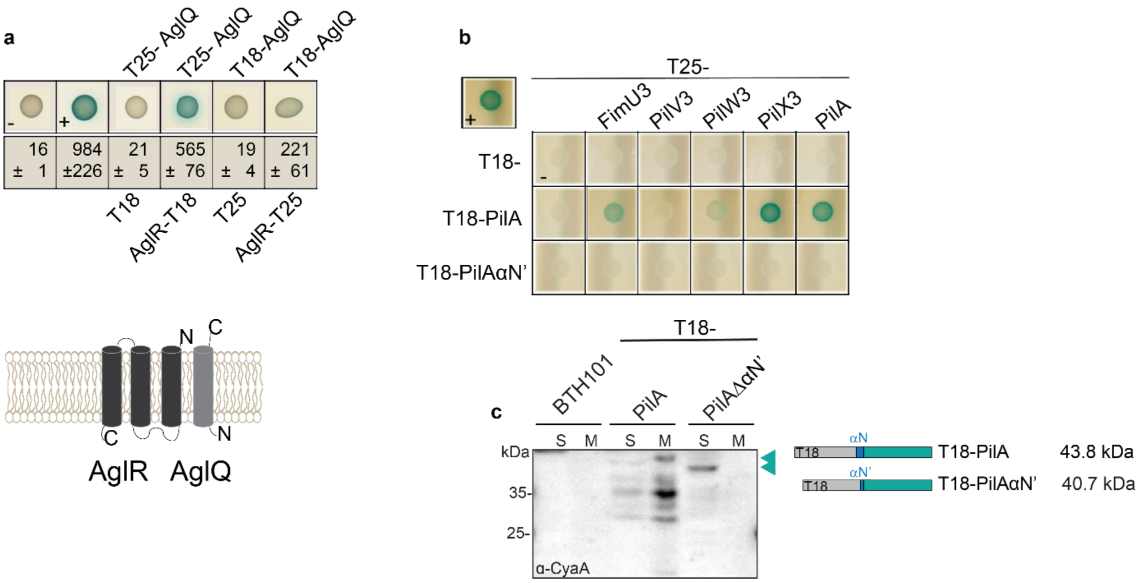

Supplementary Figure 7. Interactions between PilA and minor pilins depend on PilA localization in the inner membrane
a AglQ and AglR interact in the BACTH.
T25 and T18 were fused to the N-terminus of AglQ and to the C-terminus of AglR. Positive control (plus) T25-Zip + T18-Zip; negative control (minus), T18 + T25. For every tested interaction pair, four clones were tested with similar results. Representative images are shown of *E. coli* BTH101 containing the indicated fusions; the specific activity of β-galactosidase is shown as mean ± s.d. (n=4). Schematic, topology of the AglQ and AglR proteins in the IM[2]. **b** Interactions between PilA and cluster_3 minor pilins as well as PilA depend on the N-terminal segment of the N-terminal α-helix in PilA. T25 was fused to the N-terminus of the mature, full-length minor pilins and PilA (See Fig. 2b); T18 was fused to the N-terminus of either mature, full-length PilA (T18-PilA) or a PilA variant lacking the 28 N-terminal amino acids of the mature PilA (T18-PilAαN'), which anchor PilA in the IM (see Supplementary Fig. 1). Positive and negative controls as in (**a**). For every interaction pair, four clones were tested with similar results in two independent biological experiments. Representative images of *E. coli* BTH101 expressing the indicated protein fusions are shown. **c** Fractionation of *E. coli* BTH101 cells expressing full-length, mature PilA or the PilAα1N' variant fused to T18. Cleared cell lysates from 4×10^{10} cells were fractionated into soluble (S) and membrane (M) fractions. Protein from 1.4×10^{9} cells were loaded per lane. Blots were probed with α-CyaA. Proteins with their calculated molecular masses and positions of molecular markers are indicated. Schematics on the right, domain organization and calculated molecular sizes of the corresponding proteins. Source data are provided as a Source Data file.

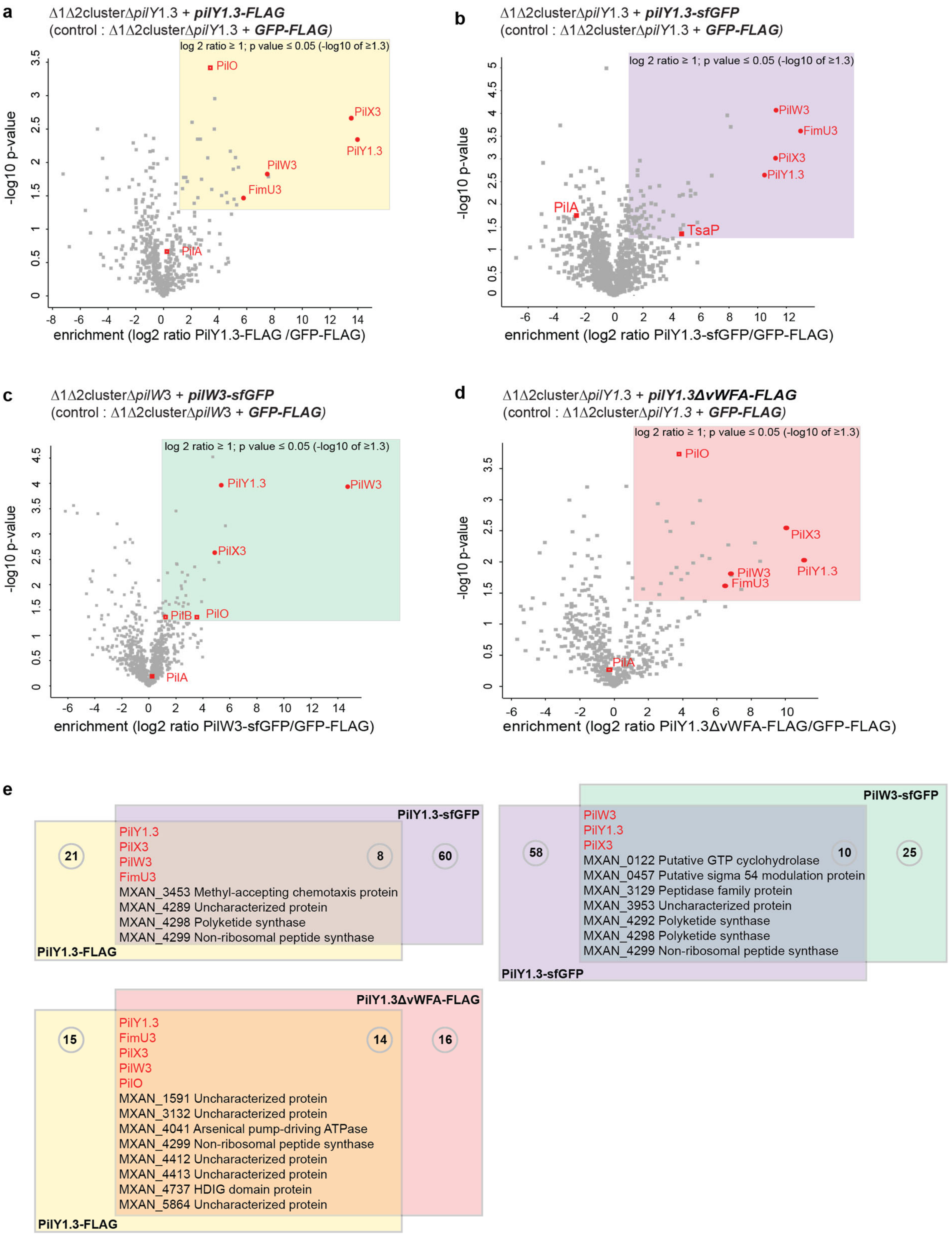

Supplementary Figure 8. Cluster_3 minor pilins and PilY1.3 interact.
a-d Volcano plots of pull-downs with tagged PilY1.3 and PilW3. For all experiments, samples from two biological replicates and two negative controls were analyzed by LFQ-MS, mean iBAQ values and log2-fold enrichment in experimental samples compared to GFP-FLAG samples calculated. X-axis, enrichment (log2 ratio) of proteins in experimental samples with

the indicated bait protein in the indicated strain versus the control sample obtained from an isogenic strain expressing a GFP-FLAG protein. Y-axis, -log10 of P-value. Significantly enriched proteins in the experimental samples (log2 ratio ≥1; P-value ≤ 0.05 (-log10 ≥1.3) are included in shaded boxes. PilY1.3, minor pilins and proteins of the T4aPM are shown in red. **e** Venn diagrams of significantly enriched proteins in pull-down experiments with the indicated bait proteins. In the pairwise comparisons, proteins known to be related to T4aPM function are indicated in red. None of the remaining proteins have been implicated in T4aPM function and with the exception of MXAN_3129, _3453 and _4737, all these proteins are predicted to be cytoplasmic. Based on sequence analyses, MXAN_3129 is predicted to be a periplasmic protein, and _3453 as well as _4737 are predicted to be inner membrane proteins. Source data are provided as a Source Data file.

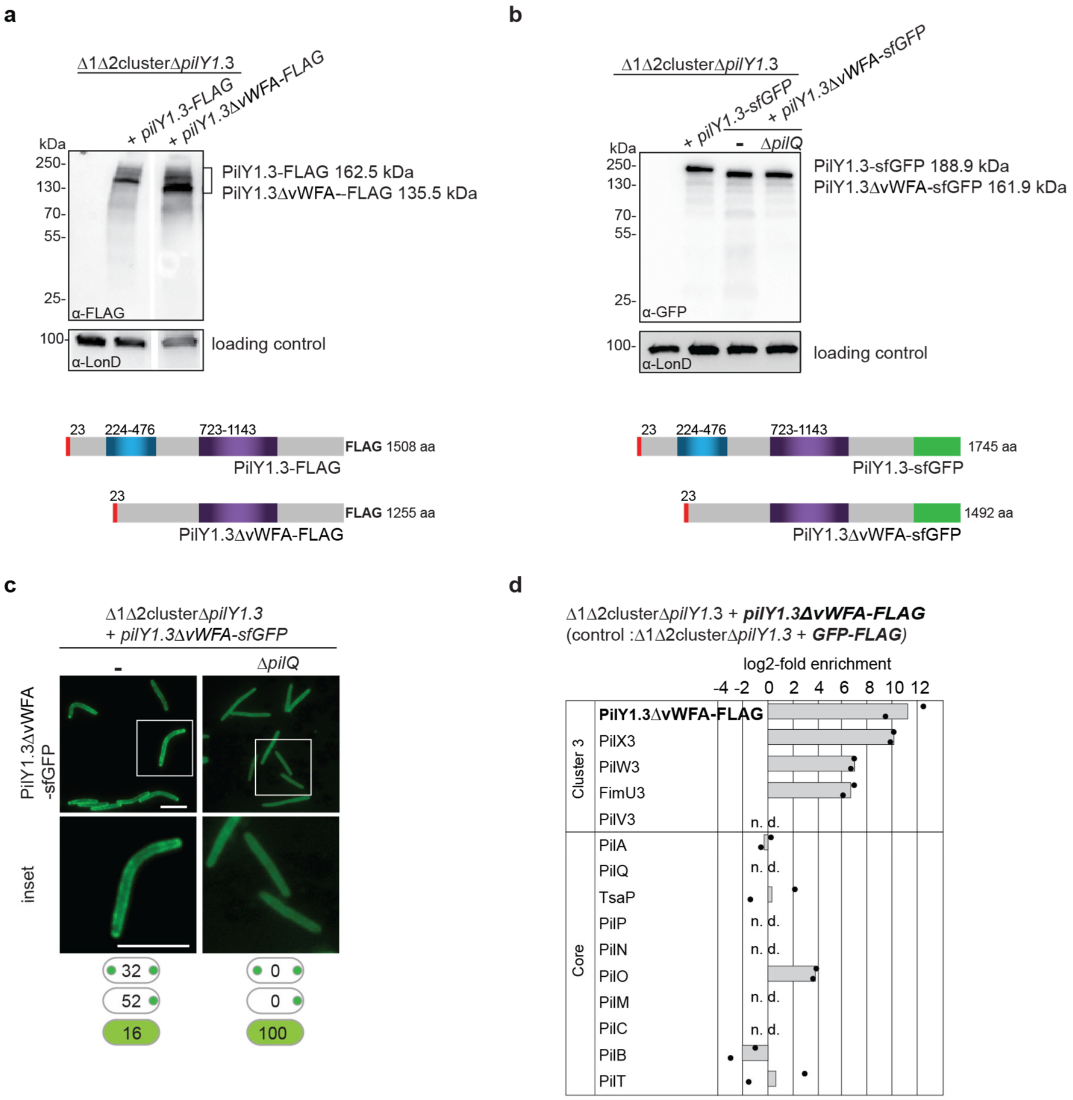

Supplementary Figure 9. PilY1.3ΔvWFA-FLAG interacts with minor pilins.
a Accumulation levels of PilY1.3ΔvWFA-FLAG. Protein from 3×10^8 cells of the indicated genotypes were loaded per lane and blots probed with α-FLAG and α-LonD antibodies (loading control). Below, the domain structures of FLAG-tagged full-length PilY1.3 and PilY1.3ΔvWFA is shown. **b** Accumulation levels of PilY1.3ΔvWFA-sfGFP. Protein from 3×10^8 cells of the indicated genotypes were loaded per lane and blots probed with α-GFP and α-LonD antibodies (loading control). Below, the domain structures of sfGFP-tagged full-length PilY1.3 and PilY1.3ΔvWFA are shown. **c** Fluorescence microscopy and analysis of mutants as in Fig. 1g. Insets are enlargements of the boxed areas. N >100 per strain; localization patterns in % indicated in schematics (bipolar, unipolar, diffuse (top-to-bottom)). Scale bars, 5 µm. Note that the fluorescent polar clusters formed by PilY1.3ΔvWFA-sfGFP were smaller and of lower intensity than those formed by full-length PilY1.3-sfGFP (Fig. 3f; Supplementary Fig. 10c). **d** PilY1.3-ΔvWFA-FLAG and cluster_3 minor pilins interact. Pull-down experiments with α-FLAG matrix on cell extracts from strain of indicated genotype expressing PilY1.3ΔvWFA-FLAG or GFP-FLAG (negative control). Samples from two biological

replicates and two negative controls were analyzed by LFQ-MS, mean iBAQ values and log2-fold enrichment in PilY1.3∆vWFA-FLAG samples compared to GFP-FLAG samples calculated; enrichment for PilY1.3-FLAG was imputed. Columns represent mean of log2-fold enrichment (n=2), dots represent the corresponding data points, n.d., not detected in PilY1.3∆vWFA-FLAG samples. Source data are provided as a Source Data file.

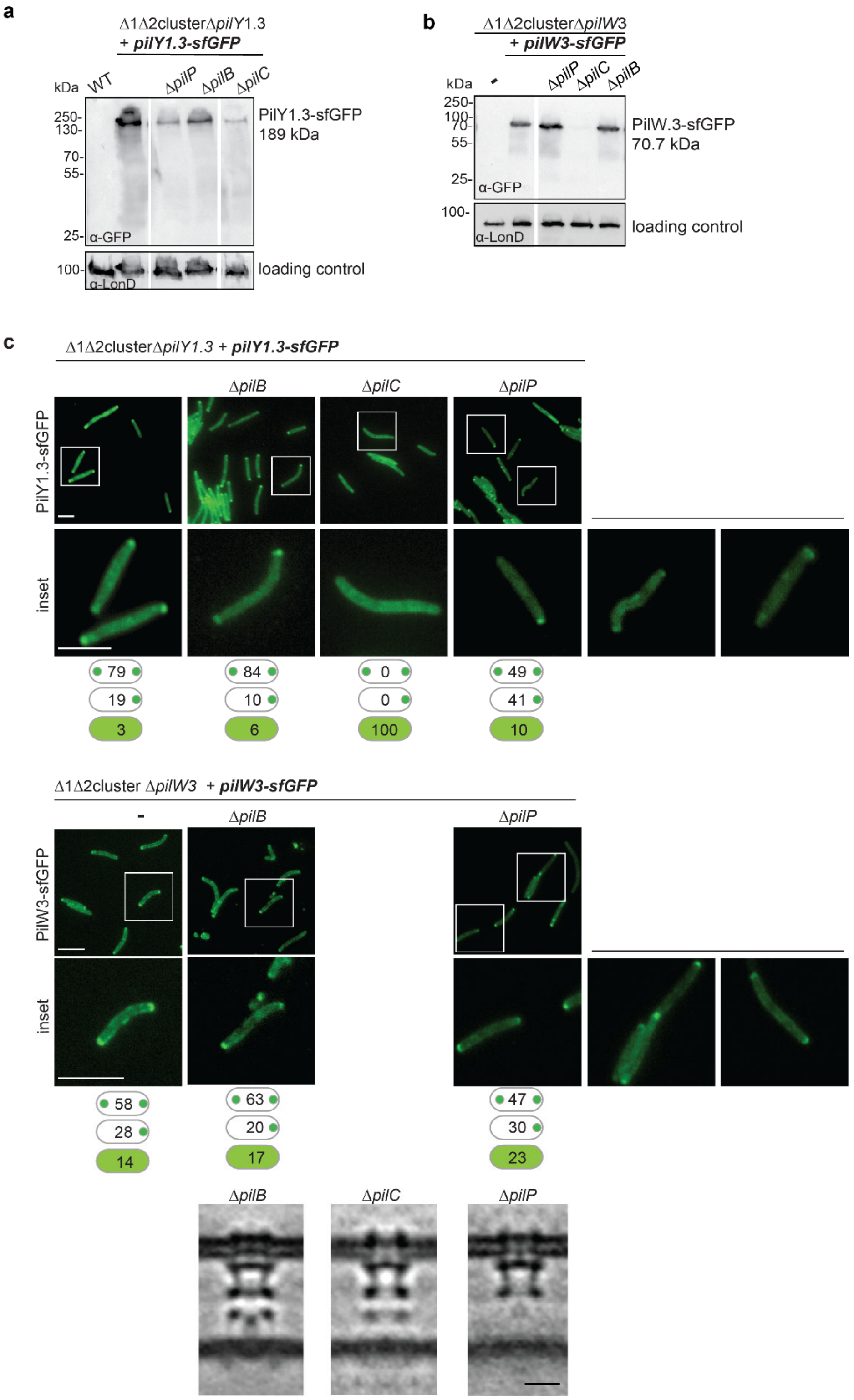

Supplementary Figure 10. PilW3-sfGFP and PilY1.3-sfGFP accumulation and localization

a, **b** Accumulation levels of PilY1.3-sfGFP (**a**) and PilW3-sfGFP (**b**) in strains of the indicated

genotypes. Protein from 3×10^8 cells were loaded per lane and blots probed with α-GFP and α-LonD antibodies (loading control). Gaps between lanes indicate lanes that were deleted for presentation purposes. **c** Correlation between PilY1.3-sfGFP and PilW3-sfGFP polar localization and presence of the short stem and plug in subtomogram averages of the non-piliated T4aPM. Fluorescence microscopy and analysis of mutants as in Fig. 1g. Insets are enlargements of the boxed areas. N >100 per strain; localization patterns in % indicated in schematics (bipolar, unipolar, diffuse (top-to-bottom)). Scale bars, 5 µm. Note that in the two ∆*pilP* strains, the fluorescent polar clusters were smaller and of lower intensity than in WT. In the ∆*pilP* mutant, the rudimentary T4aPM only contains PilQ and TsaP among the 10 core proteins[3]. Subtomogram averages of mutants with the indicated phenotype are from[3] (Reprinted with permission from AAAS). Scale bar, 10 nm. Note that fluorescence microscopy was performed on strains deleted for cluster_1 and _2 while the subtomogram averages are from mutants containing all three minor pilins/PilY1 clusters. However, the structure (subtomogram average) of the T4aPM in a strain deleted for cluster_1 and cluster_ 2 appeared essentially identical to that of WT (Fig. 4a). Source data are provided as a Source Data file.

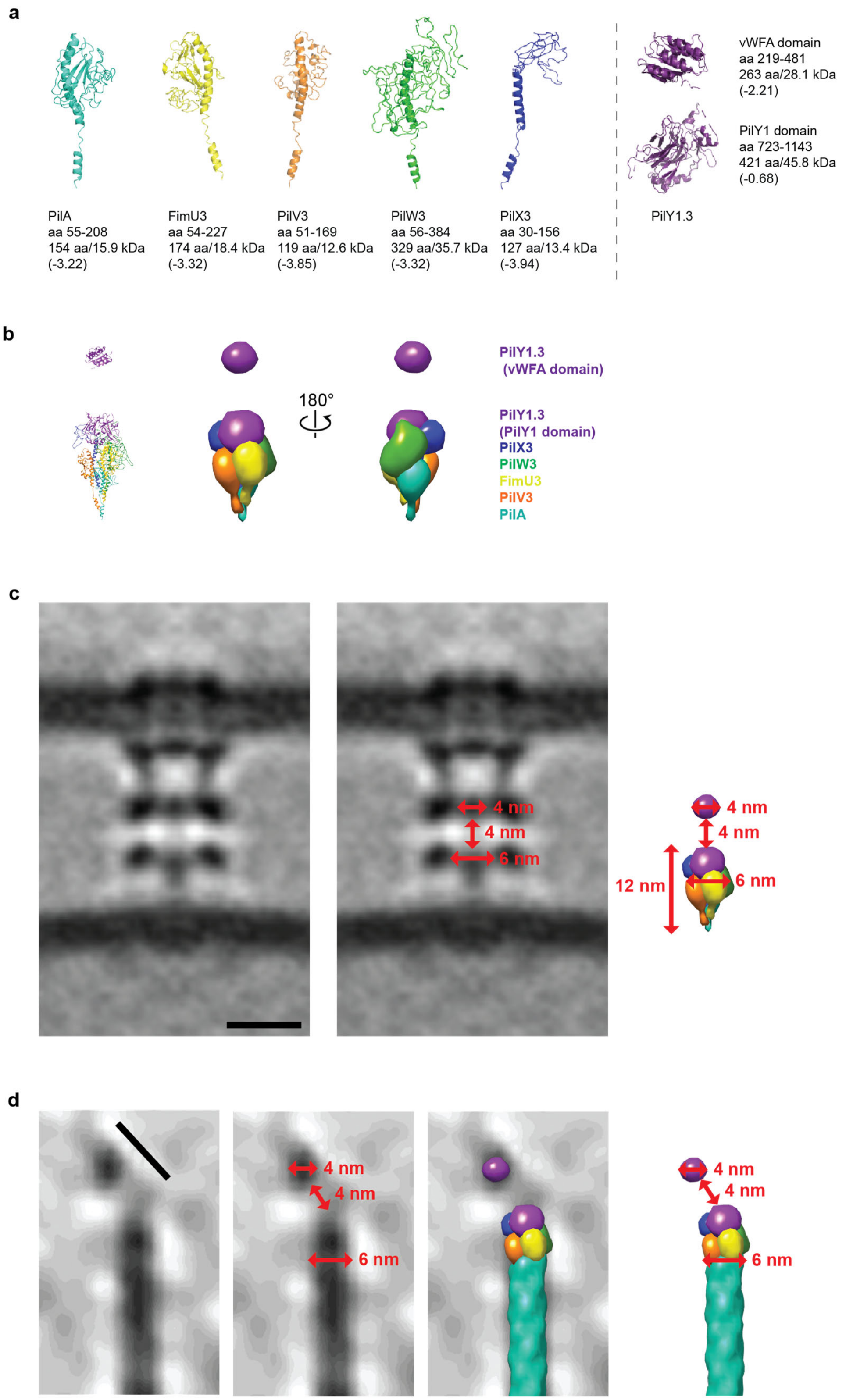

Supplementary Figure 11. Generation of hypothetical structural model of the T4aPM priming complex.

a Left panel, I-TASSER homology models were generated for the globular domains of PilA and the minor pilins of cluster_3 in *M. xanthus*. Subsequently, these models replaced the corresponding regions in the PilE structure from *N. gonorrhoeae* (5VXX)[4]. Right panel, I-TASSER homology models of the two conserved domains of *M. xanthus* PilY1.3. C-scores are expressed in numbers from -5 to +2, with +2 indicating high confidence. C-scores for the homology models are depicted below or next to the models in brackets. Amino acids included in the homology models are indicated together with their molecular masses. **b** Hypothetical structural model of priming complex shown in ribbon diagrams and molecular envelope filtered to 3 nm resolution in two orientations. Color code for individual proteins is shown on the right. **c** Dimensions of priming complex in the non-piliated WT T4aPM. Left panel, subtomogram average of WT T4aPM, scale bar, 10 nm; middle panel, subtomogram average of WT T4aPM with double-headed arrows indicating 4 and 6 nm lengths; right panel, dimensions of the hypothetical structural model of the priming complex filtered to 3 nm resolution and with color code as in **b**. **d** Dimensions of kinked pilus tip structure. 1st panel, cryo-EM image of kinked T4aP tip structure with a density gap between terminal globular density and the tip of the pilus shaft; scale bar, 10 nm. 2nd panel, the same cryo-EM image as in 1st panel with double-headed arrows indicating 4 and 6 nm lengths. 3rd panel, the same cryo-EM image as in 1st panel fitted with the hypothetical structural model of priming complex and the structure of the *N. gonorrhoeae* T4aP filament[4] together filtered to 3 nm resolution; color code as in **b**. 4th panel, hypothetical structural model of priming complex filtered to 3 nm with double-headed arrows indicating 4 and 6 nm lengths.

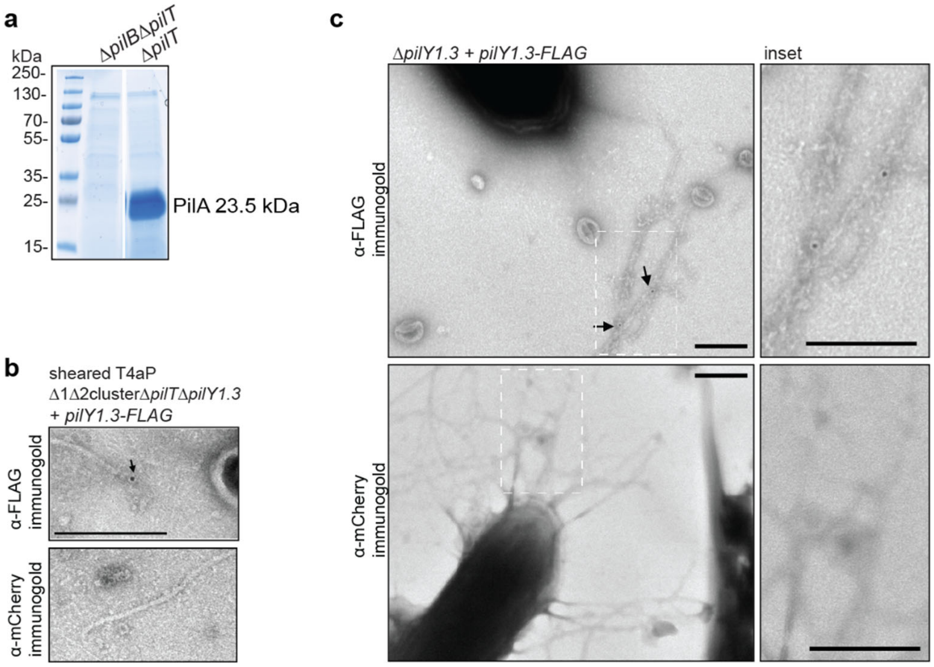

Supplementary Figure 12. Purification of T4aP and immuno-gold staining of *M. xanthus* T4aP and cells expressing PilY1.3-FLAG.

a Purification of T4aP. T4aP from the hyper-piliated Δ*pilT* strain were purified, separated by SDS-PAGE and visualized by Coomassie protein staining. As a negative control, T4aP from the same amount of non-piliated Δ*pilB*Δ*pilT* cells was loaded. Gap between lanes indicate lanes deleted for presentation purposes. **b**, **c** PilY1.3-FLAG is present in T4aP from cells containing PilY1.3-FLAG. Immuno-gold localization using rabbit α-FLAG antibodies and gold-conjugated (10 nm) sheep α-rabbit antibodies on sheared T4aP (**b**) (top panel) and cells of the indicated genotype expressing PilY1.3-FLAG (**c**) (top panel). Gold particles are indicated by arrows. Lower panels are controls in which T4aP (**b**) and cells (**c**) were first stained with rabbit α-mCherry antibodies and then with the gold-conjugated sheep α-rabbit antibodies. In **c**, the areas in the stippled boxes are shown magnified on the right. Scale bar, 200 nm. Source data are provided as a Source Data file.

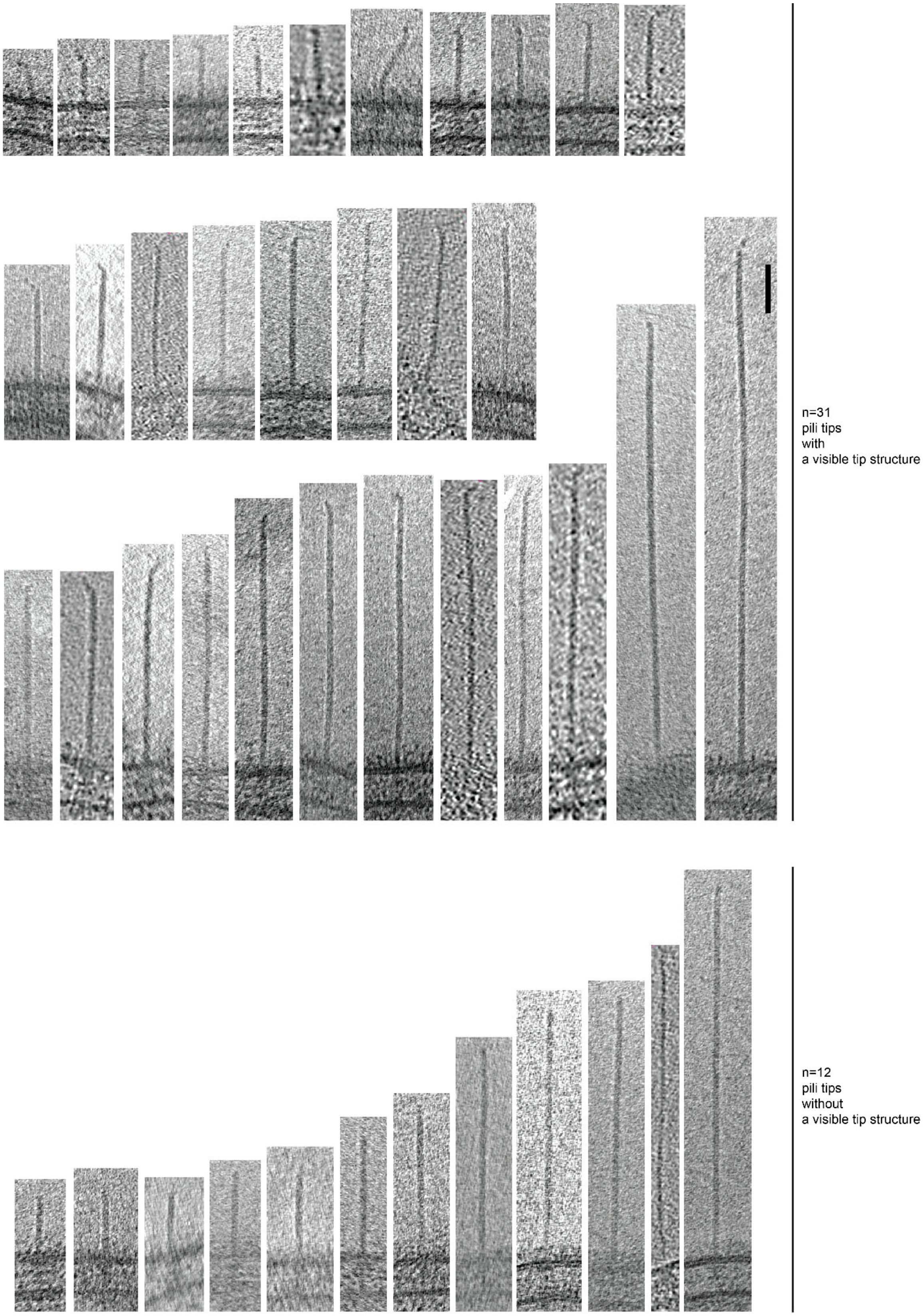
n=31
pili tips
with
a visible tip structure
n=12
pili tips
without
a visible tip structure

Supplementary Figure 13. Cryo-electron microscopy images of T4aP attached to *M. xanthus* cells

T4aP that could be traced from the cell surface to the pilus tip (n=43) sorted according to length. T4aP in which a kinked tip was visible are shown at the top and T4aP in which this structure is not visible are shown at the bottom. Scale bar, 50 nm.

Supplementary Table 1. *M. xanthus* and *E. coli* strains and plasmids used in this work

Strain	Description/Genotype[1]	Reference or source
M. xanthus		
DK1622	wildtype	[5]
SA6839	Δ1*pilY1* (ΔMXAN_0362)	This study
SA6840	Δ2*pilY1* (ΔMXAN_1020)	This study
SA6841	Δ3*pilY1* (ΔMXAN_1365)	This study
SA6842	Δ1Δ2*pilY1*	This study
SA6849	Δ1Δ3*pilY1*	This study
SA6884	Δ2Δ3*pilY1*	This study
SA6852	Δ1Δ2Δ3*pilY1*	This study
SA6850	Δ1*pilX* (ΔMXAN_0364)	This study
SA6856	Δ2*pilX* (ΔMXAN_1021)	This study
SA7604	Δ3*pilX* (ΔMXAN_1366)	This study
SA6885	Δ1Δ2*pilX*	This study
SA7605	Δ1Δ3*pilX*	This study
SA7611	Δ2Δ3*pilX*	This study
SA7607	Δ1Δ2Δ3*pilX*	This study
SA6877	Δ1cluster (ΔMXAN_0359-0364)	This study
SA6876	Δ2cluster (ΔMXAN_1021-1017)	This study
SA6891	Δ3cluster (ΔMXAN_1365-1369)	This study
SA6888	Δ1Δ2cluster	This study
SA6892	Δ2Δ3cluster	This study
SA6899	Δ1Δ3cluster	This study
SA7609	Δ1Δ2Δ3 cluster	This study
SA6794	Δ1*pilVpilWfimU* (ΔMXAN_0359-0361)	[3]
SA6819	Δ2*pilVpilWfimU* (ΔMXAN_1017-1019)	[3]
SA6796	Δ3*pilVpilWfimU* (ΔMXAN_1367-1369)	[3]
SA6818	Δ1Δ2*pilVpilWfimU*	[3]

SA6810	Δ2Δ3*pilVpilWfimU*	[3]
SA6816	Δ1Δ3*pilVpilWfimU*	[3]
SA6815	Δ1Δ2Δ3*pilVpilWfimU*	[3]
DK10410	Δ*pilA* (ΔMXAN_5783)	[6]
DK10409	Δ*pilT* (ΔMXAN_5787)	[6]
SA7667	Δ1Δ2Δ3*pilY1*Δ*pilT*	This study
SA7665	Δ1Δ2Δ3*pilX*Δ*pilT*	This study
SA7666	Δ1Δ2Δ3clusterΔ*pilT*	This study
SA7664	Δ1Δ2Δ3*pilVpilWfimU*Δ*pilT*	This study
SA7654	Δ1Δ2clusterΔ*fimU3* (ΔMXAN_1369)	This study
SA7660	Δ1Δ2clusterΔ*pilV3* (ΔMXAN_1368)	This study
SA7659	Δ1Δ2clusterΔ*pilW3* (ΔMXAN_1367)	This study
SA7655	Δ1Δ2clusterΔ*pilX3* (ΔMXAN_1366)	This study
SA7661	Δ1Δ2clusterΔ*pilY1.3* (ΔMXAN_1365)	This study
SA7894	Δ1Δ2clusterΔ*fimU3*, *attB::PpilA-fimU3* (pMAT223)	This study
SA8603	Δ1Δ2clusterΔ*pilV3*, *attB::PpilA-pilV3* (pMAT224)	This study
SA7895	Δ1Δ2clusterΔ*pilW3, attB::PpilA-pilW3* (pMAT225)	This study
SA8601	Δ1Δ2 clusterΔ*pilX3, attB::PpilA-pilX3* (pMAT338)	This study
SA7684	Δ1Δ2 clusterΔ*pilY1.3*, *attB::PpilA-pilY1.3* (pMAT212)	This study
SA8694	Δ1Δ2clusterΔ*pilV3*, *attB::PpilA-fimU3pilV3pilW3* (pMAT229)	This study
SA7718	Δ1Δ2clusterΔ*pilT*	This study
SA7690	Δ1Δ2clusterΔ*fimU3*Δ*pilT*	This study
SA7689	Δ1Δ2clusterΔ*pilV3*Δ*pilT*	This study
SA7688	Δ1Δ2clusterΔ*pilW3*Δ*pilT*	This study
SA7691	Δ1Δ2clusterΔ*pilX3*Δ*pilT*	This study
SA7709	Δ1Δ2clusterΔ*pilY1.3*Δ*pilT*	This study
SA6024	Δ*pilBTCMNOPQ*	[7]
SA6011	Δ*tsaP* (ΔMXAN_3001)	[8]
SA7896	*mCherry-pilM*	This study

SA8624	Δ1Δ2Δ3*pilVpilWfimU, mCherry-pilM*	This study
SA8625	Δ1Δ2Δ3*pilY1, mCherry-pilM*	This study
SA8685	Δ1Δ2Δ3*pilX, mCherry-pilM*	This study
SA8686	Δ1Δ2Δ3*cluster, mCherry-pilM*	This study
SA8721	Δ*pilQ, mCherry-pilM*	This study
SA3002	Δ*pilM* (ΔMXAN_5776)	[9]
SA7883	Δ1Δ2clusterΔ*pilY1.3, attB::PpilA-pilY1.3-FLAG* (pMAT209)	This study
SA7791	Δ1Δ2clusterΔ*pilY1.3, attB::PpilA-pilY1.3-sfGFP* (pMAT321)	This study
SA8674	Δ1Δ2clusterΔ*pilY1.3*Δ*pilQ, attB::PpilA-pilY1.3-sfGFP*	This study
SA7890	Δ1Δ2clusterΔ*pilY1.3, attB::PpilA-GFP-FLAG* (pMAT219)	This study
SA8704	Δ1Δ2Δ3cluster, *attB::PpilA-pilY1.3-sfGFP*	This study
SA8791	Δ1Δ2clusterΔ*pilY1.3*Δ*fimU3, attB::PpilA-pilY1.3-sfGFP*	This study
SA8797	Δ1Δ2clusterΔ*pilY1.3*Δ*pilV3, attB::PpilA-pilY1.3-sfGFP*	This study
SA8796	Δ1Δ2clusterΔ*pilY1.3*Δ*pilW3, attB::PpilA-pilY1.3-sfGFP*	This study
SA8795	Δ1Δ2clusterΔ*pilY1.3*Δ*pilX3, attB::PpilA-pilY1.3-sfGFP*	This study
SA7793	Δ1Δ2clusterΔ*pilY1.3*Δ*pilB, attB::PpilA-pilY1.3-sfGFP*	This study
SA8680	Δ1Δ2clusterΔ*pilY1.3*Δ*pilA, attB::PpilA-pilY1.3-sfGFP*	This study
SA8695	Δ1Δ2clusterΔ*pilY1.3*Δ*pilC, attB::PpilA-pilY1.3-sfGFP*	This study
SA8681	Δ1Δ2clusterΔ*pilY1.3*Δ*pilP, attB::PpilA-pilY1.3-sfGFP*	This study
SA8764	Δ1Δ2clusterΔ*pilY1.3, attB::PpilA-pilY1.3*Δ*vWFA-FLAG* (pMAT393)	This study
SA7886	Δ1Δ2clusterΔ*pilW3, attB::PpilA-pilW3-sfGFP* (pMAT332)	This study
SA11305	Δ1Δ2clusterΔ*pilW3, attB::PpilA-GFP-FLAG* (pMAT219)	This study
SA11326	Δ1Δ2clusterΔ*pilW3*Δ*pilQ, attB::PpilA-pilW3-sfGFP* (pMAT332)	This study
SA11325	Δ1Δ2clusterΔ*pilW3*Δ*pilP, attB::PpilA-pilW3-sfGFP* (pMAT332)	This study
SA11313	Δ1Δ2clusterΔ*pilW3*Δ*pilC, attB::PpilA-pilW3-sfGFP* (pMAT332)	This study
SA11310	Δ1Δ2clusterΔ*pilW3*Δ*pilB, attB::PpilA-pilW3-sfGFP* (pMAT332)	This study
SA11312	Δ1Δ2clusterΔ*pilW3*Δ*pilA, attB::PpilA-pilW3-sfGFP* (pMAT332)	This study
SA11303	Δ1Δ2clusterΔ*pilW3*Δ*pilY1.3, attB::PpilA-pilW3-sfGFP* (pMAT332)	This study

SA11339	Δ1Δ2clusterΔ*pilY1.3, attB::PpilA-pilY1.3*Δ*vWFA- sfGFP* (pMAT432)	This study
SA11344	Δ1Δ2clusterΔ*pilY1.3*Δ*pilQ, attB::PpilA-pilY1.3*Δ*vWFA- sfGFP* (pMAT432)	This study
SA7729	Δ1Δ2clusterΔ*pilY1.3*Δ*pilT attB::PpilA-pilY1.3-FLAG* (pMAT209)	This study
SA6700	Δ*pilB*Δ*pilT*	[3]
SA8728	Δ*pilY1.3 , attB::PpilA-pilY1.3-FLAG* (pMAT209)	This study
E. coli		
Mach1™-T1R	F- φ80(*lacZ*)ΔM15 Δ*lac*X74 *hsd*R(rK-mK+) Δ*rec*A1398 *endA1 tonA*	Invitrogen
Rosetta-2	F- *ompT hsdSB*(rB- mB-) *gal dcm* pRARE2 (Cam^R)	Novagen
Rosetta-2(DE3)	F- *ompT hsdSB*(rB- mB-) *gal dcm* (DE3) pRARE2 (Cam^R)	Novagen
BTH101	F- *cya*-99 *araD*139 *galE*15 *galK*16 *rpsL1* (Strr) *hsdR*2 *mcrA*1 *mcrB*1	Euromedex
**Plasmids**		
pBJ113/114	*galK* containing vector for generation of in-frame deletions in *M. xanthus*, Kan^R	[10]
pSW105	P*pilA*, Kan^R, *attP*	[11]
pSWU30	Tet^R, *attP*	[12]
pQE-30	T5 promoter, lac operator, 6x-His-tag (N-term), Amp^R	Qiagen
pET24b+	T7 promoter, lac operator, 6x-His-tag (C-term), Kan^R	Novagen
pMAT123	pBJ114, in-frame deletion construct for MXAN_5772 (Δ*pilQ* as in DK8615)	This study
pMAT150	pBJ114, in-frame deletion construct for MXAN_5787 (Δ*pilT* as in DK10409)	This study
pMAT162	pBJ114, in-frame deletion construct for MXAN_5783 (Δ*pilA* as in DK10410)	This study
pMAT163	pBJ114, in-frame deletion construct for MXAN_5788 (Δ*pilB* as in DK10416)	This study
pMAT164	pBJ114, in-frame deletion construct for MXAN_0362	This study

pMAT165	pBJ114, in-frame deletion construct for MXAN_1020	This study
pMAT166	pBJ114, in-frame deletion construct for MXAN_1365	This study
pMAT167	pBJ114, in-frame deletion construct for MXAN_0364	This study
pMAT175	pBJ114, in-frame deletion construct for MXAN_1021	This study
pMAT186	pBJ114, in-frame deletion construct for MXAN_1366	This study
pMAT168	pBJ114, in-frame deletion construct for MXAN_0359-0364, cluster 1	This study
pMAT176	pBJ114, in-frame deletion construct for MXAN_1017-1021, cluster 2	This study
pMAT187	pBJ114, in-frame deletion construct for MXAN_1369-1365, cluster 3	This study
pMAT209	pSW105 P*pilA* - *pilY1.3-FLAG*	This study
pMAT212	pSW105 P*pilA* - *pilY1.3*	This study
pMAT219	pSW105 P*pilA* – *gfp-FLAG*	This study
pMAT223	pSWU30 P*pilA* – *fimU3*	This study
pMAT224	pSWU30 P*pilA* – *pilV3*	This study
pMAT225	pSWU30 P*pilA* – *pilW3*	This study
pMAT229	pSWU30 P*pilA* – *fimU3-pilV3-pilW3*	This study
pMAT231	pQE-30 His6-*pilA* (Δ1-41aa)	This study
pMAT321	pSW105 P*pilA* – *pilY1.3-sfGFP*	This study
pMAT332	pSW105 P*pilA* – *pilW3-sfGFP*	This study
pMAT336	pBJ114,construct for endogenous *mCherry-pilM*	This study
pMAT338	pSWU30 P*pilA* – *pilX3*	This study
pMAT353	pBJ114, in-frame deletion construct for MXAN_5786 (Δ*pilC* as in DK10417)	This study
pMAT393	pSW105 P*pilA* - *pilY1.3* Δ*vWFA-FLAG*	This study
pMAT402	pBJ114, in-frame deletion construct for MXAN 1366-1365 (Δ*pilX3-pilY1.3*)	This study
pMAT432	pSW105 P*pilA* – *pilY1.3* Δ*vWFA -sfGFP*	This study
pAT2	pBJ114, in-frame deletion construct for MXAN_1367	This study

pAT3	pBJ114, in-frame deletion construct for MXAN_1368	This study
pAT4	pBJ114, in-frame deletion construct for MXAN_1369	This study
pSM30	pET24b+-*lonD*-His6 (MXAN_3993)	This study
pIB21	pBJ113, in-frame deletion construct for MXAN_5773 (PilP)	[7]
pKT25	Two-hybrid plasmid, *cyaAT25* C-terminal fusion, Kan^R	Euromedex
pUT18C	Two-hybrid plasmid, *cyaAT18* C-terminal fusion, Amp^R	Euromedex
pKNT25	Two-hybrid plasmid, *cyaAT25* N-terminal fusion, Kan^R	Euromedex
pUT18	Two-hybrid plasmid, *cyaAT18* N-terminal fusion, Amp^R	Euromedex
pKT25-zip	Two-hybrid control plasmid	D. Ladant (Euromedex)
pUT18C-zip	Two-hybrid control plasmid	D. Ladant (Euromedex)
p18C-PilA-SP	pUT18C, *cyaT18*-PilA-SP(Δ1-12aa)	This study
pKT-PilA-SP	pKT25, *cyaT25*-PilA-SP(Δ1-12aa)	This study
p18C-PilA-TM	pUT18C, *cyaT18*-PilA-TM(Δα1N; Δ1-41aa)	This study
pKT-PilA-TM	pKT25, *cyaT25*-PilA-TM(Δα1N; Δ1-41aa)	This study
p18C-FimU3-SP	pUT18C, *cyaT18*-FimU3-SP(Δ1-8aa)	This study
pKT-FimU3-SP	pKT25, *cyaT25*-FimU3-SP(Δ1-8aa)	This study
p18C-PilV3-SP	pUT18C, *cyaT18*-PilV3-SP(Δ1-15aa)	This study
pKT-PilV3-SP	pKT25, *cyaT25*-PilV3-SP(Δ1-15aa)	This study
p18C-PilW3-SP	pUT18C, *cyaT18*-PilW3-SP(Δ1-11aa)	This study
pKT-PilW3-SP	pKT25, *cyaT25*-PilW3-SP(Δ1-11aa)	This study
p18C-PilX3-SP	pUT18C, *cyaT18*-PilX3-SP(Δ1-9aa)	This study
pKT-PilX3-SP	pKT25, *cyaT25*-PilX3-SP(Δ1-9aa)	This study
p18C-AglQ	pUT18C, *cyaT18*-AglQ	This study
pKT-AglQ	pKT25, *cyaT25*-AglQ	This study
p18-AglR	pUT18, *cya AglR-T18*	This study
pKNT-AglR	pKTN25, *cya AglR-T25*	This study

[1] Plasmids for ectopic expression of genes in *M. xanthus* are all derivatives of pSW105 and pSWU30 and were integrated by site specific recombination at the chromosomal *attB* site. Plasmids names are listed in parentheses. P*pilA* indicates that these genes were expressed from the *pilA* promoter. Coordinates for major and minor pilins corresponds to the full-length proteins, before cleavage by the PilD prepilin peptidase.

Supplementary Table 2. Oligonucleotides used in this work

Name	Sequence[1]
mxan_0362-A BamHI	**GCGCGGATCC**CATCCGGCTGGGCAATCTGC
mxan_0362-B overlay	CCGGGCTGGGGGCGGCCAGTGTCTGGATGAG
mxan_0362-C overlay	CAGACACTGGCCGCCCCCAGCCCGGACGGG
mxan_0362-D HindIII	**GCGCAAGCTT**TTCTCAACCGTGCAGAACGTCC
mxan_0362-E	CAAGGAGGCCTACTTCGCGG
mxan_0362-F	CGTTGGTGACTTCAACATGG
mxan_0362-G	CCATGTCTGAAGCAGGACCC
mxan_0362-H	GTTGGCCAGCGCGGACGGTG
mxan_0364-A HindIII / cluster 1-A	**GCGCAAGCTT**GGTGAAGGACCTCATCGAG
mxan_0364-B XbaI / cluster 1-B	**GCGCTCTAGA**CCGCCGTCGGGTTGTGGG
mxan_0364-C XbaI	**GCGCTCTAGA**GGAAATGAAGTGGACATC
mxan_0364-D BamHI	**GCGCGGATCC**CATAGCCCCTGACGCGGAGG
mxan_0364-E / cluster 1-E	GAATGACTTCCTGTCTCCCG
mxan_0364-F	GTCGACAGCAACAACAACGG
mxan_0364-G	CATGGGCGTCGTCACGATGG
mxan_0364-H	GCCGGAGCCGTCGAAGGTGC
mxan_0359-C XbaI / cluster 1-C	**GCGCTCTAGA**TGAGCACTGCCGGCACCTGAAG
mxan_0359-D BamHI / cluster 1-D	**GCGCGGATCC**CGGAGGTGGAGCTGCTGC
mxan0361-0359 F/ cluster 1-F	CTTCGACCCGGCGAAGCACG
mxan_1020-A BamHI	**GCGCGGATCC**CGTCCCACTTTGTCGCTGAC
mxan_1020-B overlay	GCGTGAGCCGGACGCGGCCATCCAGGTTCG
mxan_1020-C overlay	TGGATGGCCGCGTCCGGCTCACGCGTCCAG
mxan_1020-D HindIII	**GCGCAAGCTT**GAAGGCGGGCGGCAGGCCTC
mxan_1020-E	CTGTCACGGAGCATCCACCC
mxan_1020-F	CCACGCCTTCCGCAACGAGG
mxan_1020-G	CTGGGACAAATCCGATGTGG
mxan_1020-H	CACCCGGGCTCTCCATCACC

mxan_1021-A AseI / cluster 2-A	**GCGC<u>ATTAAT</u>**GGAGCCAGTCGCCGGAAGCC
mxan_1021-B XbaI / cluster 2-B	**GCGC<u>TCTAGA</u>**TGCGGGCTTGCGGGTCGGATG
mxan_1021-C XbaI	**GCGC<u>TCTAGA</u>**GGCACCTCCAGCAGCGGCC
mxan_1021-D EcoRI	**GCGC<u>GAATTC</u>**CCGATTCCGATTCGTGGCAC
mxan_1021-E / cluster 2-E	CTGTTCACCCTGGCGCCC
mxan_1021-F	CATACAGGTACACGCTG
mxan_1021-G	GACCACGGAGAGCACGCTCC
mxan_1021-H	GCTCCAGGAGTGAGCGGACG
mxan_1017-C XbaI / cluster 2-C	**GCGC<u>TCTAGA</u>**TAGGCGGCGTCCGCACCG
mxan_1017-D EcoRI / cluster 2-D	**GCGC<u>GAATTC</u>**GCCGGTGGCCTGCTGCTAGC
Mxan1019-1017 F/ cluster 2-F	GGGCCGCCAGGCAAGACTGC
mxan_1365-A BamHI	**GCGC<u>GGATCC</u>**CGCCGACTTCGACCCCATCG
mxan_1365-B overlay	GTTCTCGTGGCGGCTGTCAGGGTGGAGAA
mxan_1365-C overlay	ACCCTGACAGCCCGCCACGAGAACGCGGCC
mxan_1365-D HindIII	**GCGC<u>AAGCTT</u>**CGGATGAGCATCTACGACGC
mxan_1365-Enew / cluster 3-E	CATGCTGGTGCATGAGAAGC
mxan_1365-Fnew / cluster 3-F	CGGCAACCACGAGGTGATGG
mxan_1365-Gnew	CAGGCGGTGGCCACCTACAC
mxan_1365-Hnew	CCGGAGGCCCGTCATCAACG
mxan_1366-A HindIII	**GCGC<u>AAGCTT</u>**GGACTACTTCCTCGGGCGGC
mxan_1366-B XbaI	**GCGC<u>TCTAGA</u>**GCTGCCCCGGCGTGAGGGC
mxan_1366-C XbaI	**GCGC<u>TCTAGA</u>**GAGACCGAGTTCGTCTTC
mxan_1366-D BamHI	**GCGC<u>GGATCC</u>**GCGCACCACGTCCTGGCCGC
mxan_1366-Enew	GCCCTTCCAGTTGGAGCTGG
mxan_1366-Fnew	GTGATCTTCACGCTTCGCGC
mxan_1366-G	GCCCTGGCCATCACCCTG
mxan_1366-H	GCCTCCGGGTTCGTCACA
mxan_1369-A HindIII / cluster 3-A	**GCGC<u>AAGCTT</u>**GTTGTCCGTCAGCGCCACCG
mxan_1369-B XbaI / cluster 3-B	**GCGC<u>TCTAGA</u>**CCGGCTCACTTGGCGACCTCC

mxan_1365-C XbaI / cluster 3-C	**GCGC<u>TCTAGA</u>**CGCCACGAGAACGCGGCC
mxan_1365-D BamHI / cluster 3-D	**GCGC<u>GGATCC</u>**CGGATGAGCATCTACGACGC
pilA(Δ1-123)-BamHI-factor Xa fwd	**GCGC<u>GGATCC</u>**GACATCGAGGGCCGCCGCTCGAAGCAGTCCGAG
pilA stop HindIII	**GCGC<u>AAGCTT</u>**TTACTGGGCCGCGCCGTCGCAGGCGAC
LonD-overex. fwd NdeI (MS-81)	**ATCG<u>CATATG</u>**ATGTCCGATGAGAAGAAGAAG
LonD-overex. rev no stop HindIII (MS-82)	**ATCG<u>AAGCTT</u>**GGCGCGGACCTCAGGGGCGG
pilT-A EcoRI	**GCGC<u>GAATTC</u>**CGCGACTTCGAGACGGCGG
pilT-D HindIII	**GCGC<u>AAGCTT</u>**GAGCTTCTCGTTCTTCTCC
5787-G pilT	CTTGAAGACGGCGCCGCTGA
5787-H pilT	CGCGCTGATTCACGAGGCAG
pilT-E	CTCCGCCAGGACCCGGACATC
pilT-F- (also used as pilB-F)	CGAAGACGGGCGTCACCTTC
mxan_1369-C XbaI	**GCGC<u>TCTAGA</u>**GTCAACCGATGAGGCGCCTG
mxan_1369-D BamHI	**GCGC<u>GGATCC</u>**CCTGCGTGTAGTTGTCCTTG
mxan_1368-A HindIII	**GCGC<u>AAGCTT</u>**AGCCGAATCAAATCATGGTC
mxan_1368-B XbaI	**GCGC<u>TCTAGA</u>**GCGCCTCATCGGTTGACCCT
mxan_1368-C XbaI	**GCGC<u>TCTAGA</u>**GTGGAGCTGTGAACGCCCCC
mxan_1368-D BamHI	**GCGC<u>GGATCC</u>**GAACGTGGTCGTCGTACTTG
mxan_1367-A HindIII	**GCGC<u>AAGCTT</u>**CTGGCGTGGACCGGCATCTT
mxan_1367-B XbaI	**GCGC<u>TCTAGA</u>**GGCGTTCACAGCTCCACCCC
mxan_1367-C XbaI	**GCGC<u>TCTAGA</u>**TAGCCATCATGACCCACTTC
mxan_1367-D BamHI	**GCGC<u>GGATCC</u>**CGCTGCCATCGAAGCGGTAG
mxan_1369 start XbaI	**GCGC<u>TCTAGA</u>**GTGAGCCGGCGCGACGCAC
mxan_1369 stop HindIII	**GCGC<u>AAGCTT</u>**TCATCGGTTGACCCTCATG
mxan_1368 start XbaI	**GCGC<u>TCTAGA</u>**ATGAGGCGCCTGAATCCC
mxan_1368 stop HindIII	**GCGC<u>AAGCTT</u>**TCACAGCTCCACCCCCGC
mxan_1367 start XbaI	**GCGC<u>TCTAGA</u>**GTGAACGCCCCCAGGACGC
mxan_1367 stop HindIII	**GCGC<u>AAGCTT</u>**CTAGCCTCCCCAGACGTTC

mxan_1366 start XbaI	**GCGCTCTAGA**ATGACCCACTTCGCCTCAC
mxan_1366 stop HindIII	**GCGCAAGCTT**CTAGCCTCCCCAGACGTTC
mxan_1365 start XbaI	**GCGCTCTAGA**ATGAAGGCACTCTTCTCCACC
mxan_1365 stop HindIII	**GCGCAAGCTT**TCACGGCAGGCAACTGGCCG
pilM-prom fwd EcoRI	**GCGCGAATTC**CCTGAAGTCCTACGCATG
pilM-mCherry overlay reverse	**CATACTAGTAGATCT**GCGTGACTCCGTCGAGAG
mCherry+linker overlay forward	**AGATCTACTAGT**ATGGTGAGCAAGGGCGAGGAG
mCherry+linker-overlay reverse	**CAGTTTGCCCTTCGC**TCTAGAGGATCCCCGGGTAC
pilM-overlay forward	GCGAAGGGCAAACTGGTACTCGGC
pilM int rev HindIII	**GCGCAAGCTT**GATGTTCACCACCGAGGC
pilM-E(pMAT336)	GAGCCTTCATCGCTCGG
pilM-F(pMAT336)	CGGTGAACTGGTTGCCG
B2H-pilA-SP fwd XbaI	**GCGCTCTAGAG**TTCACGCTCATCGAGCTCATGATCGTG
B2H-pilA no stop BamHI	**CGCGGATCCGG**CTGGGCCGCGCCGTCGCAGG
B2H-fimU3-SP fwd XbaI	**GCGCTCTAGAG**ATGACGCTGCTGGAGGTC
B2H-fimU3 no stop BamHI	**CGCGGATCCGG**TCGGTTGACCCTCATGTC
B2H-pilV3-SP fwd XbaI	**GCGCTCTAGAG**GCCACGCTCATCGAAGGC
B2H-pilV3 no stop BamHI	**CGCGGATCCGG**CAGCTCCACCCCCGCGCC
B2H-pilW3-SP fwd XbaI	**GCGCTCTAGAG**TTCACGCTCATCGAGTTG
B2H-pilW3 no stop BamHI	**CGCGGATCCGG**GCCTCCCCAGACGTTCTT
B2H-pilX3-SP fwd XbaI	**GCGCTCTAGAG**AGCGCCCTGGCCATCACC
B2H-pilX3 no stop BamHI	**CGCGGATCCGG**GATGCCGAAGCGGAAGAC
B2H-pilA-TM fwd XbaI	**GCGCTCTAGAG**CGCTCGAAGCAGTCCGAGG
mxan_1365 no stop BamHI	**GCGCGGATCC**CGGCAGGCAACTGGCCGCGTTC
XI-S-B-(flag) stop HindIII fwd	CTAGAACTAGTGGATCC(GACTACAAGGACGACGACGACAAG)TGA
XI-S-B-(flag) stop HindIII rev	AGCTTCA(CTTGTCGTCGTCGTCCTTGTAGTC)GGATCCACTAGTT
GFP-XbaI start(2nd codon)	**GCGCTCTAGA**GCCAAGGGCGAGGAGCTG
GFP-BamHI no stop	**GCGCGGATCC**CTTGTACAGCTCGTCCATG

pilQ-A-EcoRI	**GCGC<u>GAATTC</u>**GTCGCCGATGGTCGAAGACCC
pilQ-D-BamHI	**GCGC<u>GGATCC</u>**GGTGCGTGCGGAAGGACATG
pilQ-E	CGACTTGGATCAGCTGAAGC
pilQ-F	CTGCACGCGAGGGCTCCTTC
pilQ-G	GCGCCCGCTCGTTGGTGCGC
pilQ-H	CACCTCCAACGACAGACGCG
linker-sfGFP-BamHI fwd	**GCGC<u>GGATCC</u>**CTGGAGGGCCCGGCGGGCCTGATGAGCAAAGGAGAAGAAC
sfGFP-HindIII reverse	**GCGC<u>AAGCTT</u>**TTATTTGTAGAGCTCATCC
pilB-A EcoRI	**GCGC<u>GAATTC</u>**CACCCTGCTGCCGCGCAAGC
pilB-D HindIII	**GCGC<u>AAGCTT</u>**CTGGTTGAAGGTCTGCATGC
pilB-E2	CAGGCAAGGTGCTCCAGCCG
pilC-A EcoRI	**GCGC<u>GAATTC</u>**CGCTCACCATCGCGGAGACG
pilC-D HindIII	**GCGC<u>AAGCTT</u>**GCCCAGGGCCAGGGAGTTGC
pilC-E	GTGACCTGGAGACGATTG
pilC-F	GGAGAGCTGACGTGAGAG
pilC-G	GCCTCGATATCCTCGCGAG
pilC-H	GATGAGGAAGCCACCGACC
pilA-A EcoRI	**GCGC<u>GAATTC</u>**CACTGGCGCGACCACCGACC
pilA-D HindIII	**GCGC<u>AAGCTT</u>**GAACTGAATGCCACCCGCCG
pilA-E	CGCTTCCGGCCGCAGCACGG
pilA-F2	CAGCAGTCCGTAGACCTGGC
pilA-G	CCTGGCCGCCATCGCCATCC
pilA-H	CGATCACCCAGTCATCGAAG
B2H-aglR-F HinDIII	**CCCAAGCTTG**GACCTGGCGTCTGTGAC
B2H-aglR-R EcoRI	**CCCGGAATTCTC**CTCCTCGTCGCGAGCCG
B2H-aglQ-F-XbaI	**GCGCTCTAGAG**ATGGCCGGCGGAATGGAC
B2H-aglQ-R-BamHI	**GCGCGGATCCTC**GCCCATCGCCGCGGACAC
delta vWFA+	TCGCCGCGCGGCCACGTGCAGAACCGGAAATC

delta vWFA-	GTTCCGGTTCTGCACGTGGCCGC CGGCGAATA
pilW3-no stop-BamHI	**GCGCGGATCC**GCCTCCCCAGACGTTCTTG

[1] Primer sequences that are not complementary to the template are indicated in bold. Restriction sites are underlined.

Supplementary References

1 Orans, J. *et al.* Crystal structure analysis reveals *Pseudomonas* PilY1 as an essential calcium-dependent regulator of bacterial surface motility. *Proc. Natl. Acad. Sci. USA* **107**, 1065-1070, doi:10.1073/pnas.0911616107 (2010).

2 Sun, M., Wartel, M., Cascales, E., Shaevitz, J. W. & Mignot, T. Motor-driven intracellular transport powers bacterial gliding motility. *Proc Natl Acad Sci U S A* **108**, 7559-7564, doi:10.1073/pnas.1101101108 (2011).

3 Chang, Y. W. *et al.* Architecture of the type IVa pilus machine. *Science* **351**, aad2001, doi:10.1126/science.aad2001 (2016).

4 Wang, F. *et al.* Cryoelectron microscopy reconstructions of the *Pseudomonas aeruginosa* and *Neisseria gonorrhoeae* type IV pili at sub-nanometer resolution. *Structure* **25**, 1423-1435 e1424, doi:10.1016/j.str.2017.07.016 (2017).

5 Kaiser, D. Social gliding is correlated with the presence of pili in *Myxococcus xanthus*. *Proc. Natl. Acad. Sci. USA* **76**, 5952-5956 (1979).

6 Wu, S. S., Wu, J. & Kaiser, D. The *Myxococcus xanthus pilT* locus is required for social gliding motility although pili are still produced. *Mol. Microbiol.* **23**, 109-121 (1997).

7 Friedrich, C., Bulyha, I. & Søgaard-Andersen, L. Outside-in assembly pathway of the type IV pilus system in *Myxococcus xanthus*. *J. Bacteriol.* **196**, 378-390, doi:10.1128/JB.01094-13 (2014).

8 Siewering, K. *et al.* Peptidoglycan-binding protein TsaP functions in surface assembly of type IV pili. *Proc. Natl. Acad. Sci. USA* **111**, E953-961, doi:10.1073/pnas.1322889111 (2014).

9 Bulyha, I. *et al.* Regulation of the type IV pili molecular machine by dynamic localization of two motor proteins. *Mol. Microbiol.* **74**, 691-706, doi:10.1111/j.1365-2958.2009.06891.x (2009).

10 Julien, B., Kaiser, A. D. & Garza, A. Spatial control of cell differentiation in *Myxococcus xanthus*. *Proc. Natl. Acad. Sci. USA* **97**, 9098-9103 (2000).

11 Jakovljevic, V., Leonardy, S., Hoppert, M. & Søgaard-Andersen, L. PilB and PilT are ATPases acting antagonistically in type IV pilus function in *Myxococcus xanthus*. *J. Bacteriol.* **190**, 2411-2421, doi:10.1128/JB.01793-07 (2008).

12 Wu, S. S. & Kaiser, D. Regulation of expression of the *pilA* gene in *Myxococcus xanthus*. *J. Bacteriol.* **179**, 7748-7758 (1997).
